# Extranodal Marginal Zone Lymphoma Presenting within the Meckel Diverticulum as Diverticulitis: A Case Report

**DOI:** 10.1155/2014/374814

**Published:** 2014-04-28

**Authors:** A. Nael, M. L. Wu, P. Nagesh Rao, S. Rezk, X. Zhao

**Affiliations:** ^1^Department of Pathology and Laboratory Medicine, University of California Irvine Medical Center, 101 The City Drive, Orange, CA 92868, USA; ^2^Clinical Pathology, University of California Irvine Medical Center, 101 The City Drive, Orange, CA 92868, USA; ^3^Pathology and Lab Medicine and Pediatrics, David Geffen University of California Los Angeles School of Medicine, 1000 Veteran Avenue, Rm 22-26, Los Angeles, CA 90095, USA; ^4^Department of Pathology, University of California Irvine Medical Center, 101 The City Drive, Orange, CA 92868, USA

## Abstract

Meckel diverticulum is the most common congenital defect of the gastrointestinal tract. It can be asymptomatic or mimic appendicitis and may be complicated by bleeding, diverticulitis, obstruction, and, rarely, neoplasia. We report the first case of extranodal marginal zone lymphoma occupying a Meckel diverticulum. A 44-year-old man with history of colonic diverticulitis presented to the emergency department for evaluation of acute abdominal pain. Radiography showed enteric obstruction, prompting diagnostic laparoscopy. Above the level of mid-ileum an intact Meckel diverticulum was identified. Microscopy showed extensive infiltration of sheets of small lymphocytes with abundant cytoplasm (monocytoid B-cells) prominently in submucosa and focally transmural involving serosal adipose tissue with multiple reactive germinal centers. The immunostains showed positivity for CD20, BCL-2, and CD43 (weak) and negativity for CD3, CD5, BCL-1, CD10, and BCL-6 in monocytoid B-cells. Fluorescence in situ hybridization studies revealed API2-MALT1 fusion signals consistent with t(11;18)(q21;q21), which confirmed the diagnosis of extranodal marginal zone lymphoma, also known as mucosa associated lymphoid tissue lymphoma.

## 1. Introduction

Meckel diverticulum (MD) is the most common congenital defect of the gastrointestinal (GI) tract. It is a part of vitelline duct, which connects the growing fetus with the yolk sac. When the vitelline duct is not fully absorbed, a MD develops in the lower part of small intestine. Histologically, it is a true diverticulum, containing all tunicae of GI tract and may or may not contain ectopic gastric or pancreatic epithelium. MD can be asymptomatic or mimic appendicitis clinically or may be complicated by bleeding, diverticulitis, obstruction, perforation, and, rarely, neoplasia [1]. Malignant tumors arising from MD are rare and there are a few reported cases of carcinoid tumors, gastrointestinal stromal tumor (GIST), signet ring cell carcinoma, and adenocarcinoma [2–4]. Lymphomas occurring in MD are exceedingly rare and there are only three cases in the English literature including Burkitt's lymphoma, plasmacytoid lymphoblastic lymphoma, and one B-cell lymphoma with no further classification [5–8]. We report the first case of extranodal marginal zone lymphoma (ENMZL) arising in a MD. The method of choice for diagnosis of MD in children is a technetium-99 m (99 mTc) scan, which detects gastric mucosa. Since about 50% of symptomatic MD cases have gastric mucosa, it is highly accurate and noninvasive, with 95% specificity and 85% sensitivity; however, its sensitivity and specificity in adults are low [9]. Although other imaging studies or even colonoscopy would be helpful in diagnosis, in many cases, a laparoscopy is necessary to confirm the diagnosis. Finally, surgical resection is the treatment of choice for symptomatic MD but the treatment of asymptomatic cases encountered at laparotomy remains controversial [10].

## 2. Case Presentation

**Figure 1:**
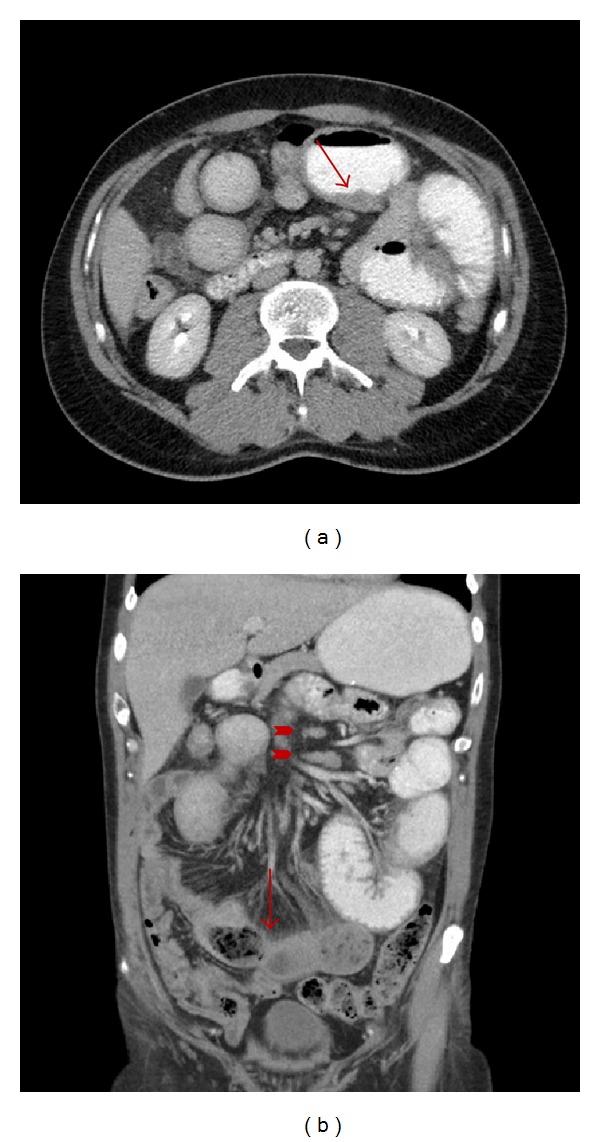
Axial (a) and coronal (b) CT images obtained after IV and oral contrast administration are shown. Note asymmetric wall thickening involving some of the ileal bowel loops (arrow in (a)). There is dilation of several small bowel loops with a transition point in the mid-lower quadrant (arrow in (b)) consistent with small bowel obstruction. There are also a few borderline enlarged mesenteric lymph nodes (arrowheads in (b)).

**Figure 2:**
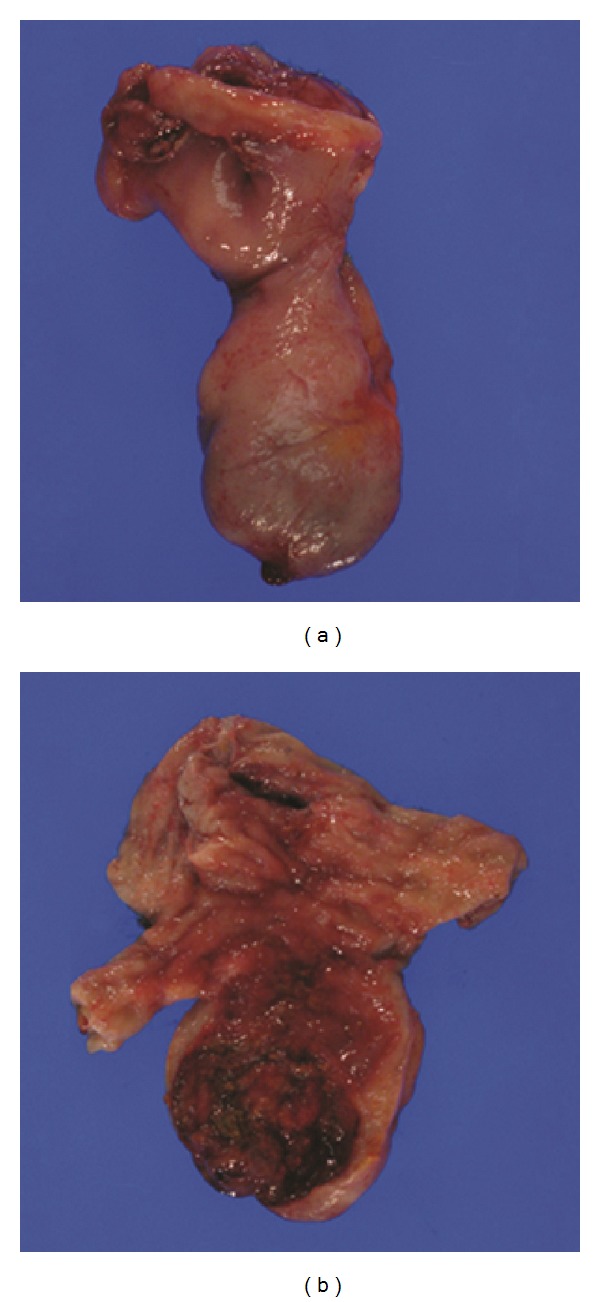
Meckel diverticulum gross pictures before and after dissection to show (a) intact diverticulum and (b) mucosal ulcer with severe edema and erythema.

**Figure 3:**
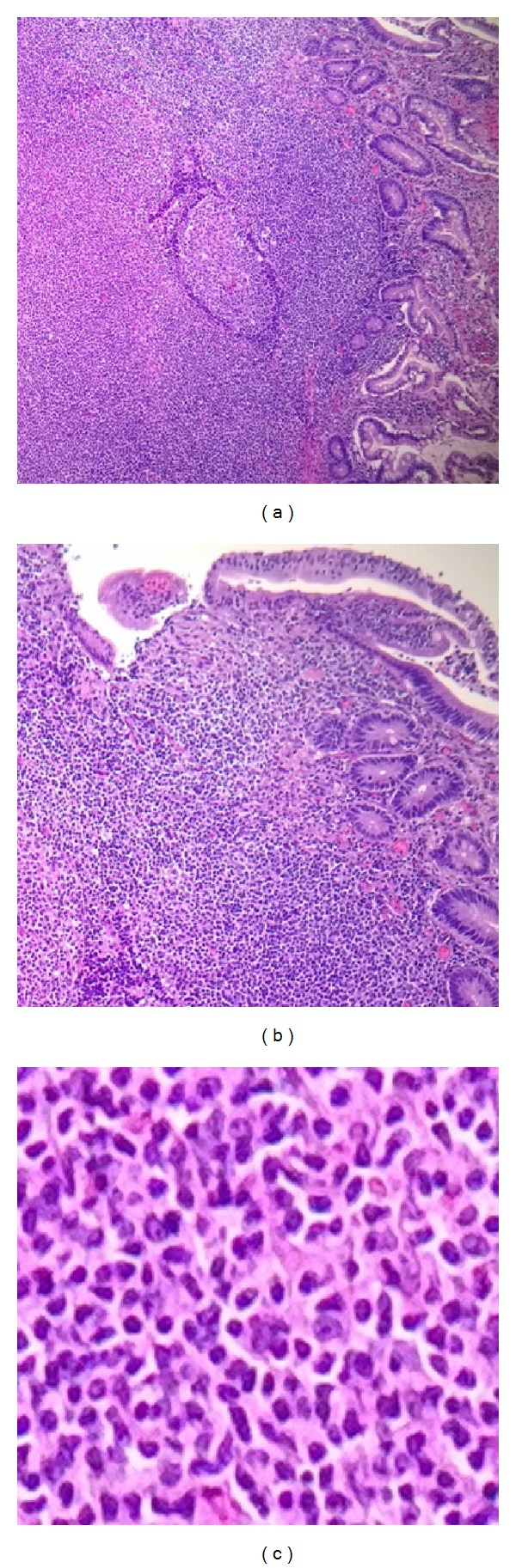
Microscopic pictures of Meckel diverticulum to show (a) sheets of infiltrative small lymphocytes involving mucosa and submucosa, (b) epithelial erosion and lymphoepithelial lesion, and (c) small lymphocytes with irregular nuclear contour and abundant cytoplasm (monocytoid B-cells). (hematoxylin-eosin, original magnification ×40 (a), ×100 (b), and ×400(c)).

**Figure 4:**
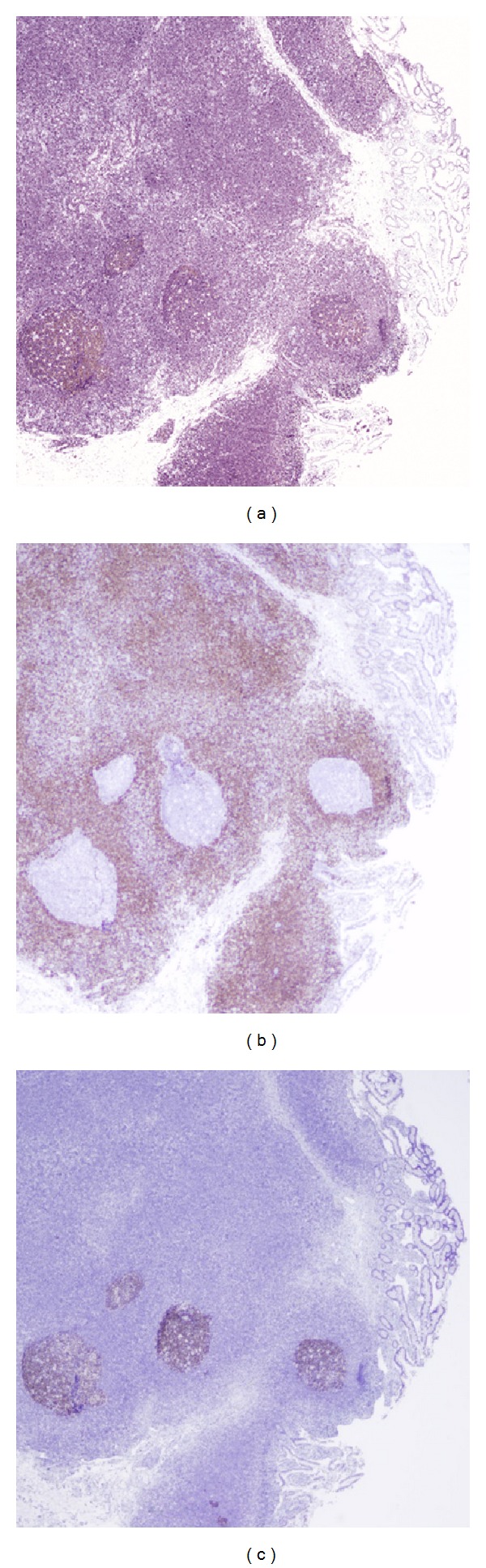
Microscopic pictures from Meckel diverticulum show (a) Diffuse positivity for CD20; (c) and (d) reactive germinal centers with negativity for BCL-2 (b) and positivity for BCL-6 (c) (original magnification ×100 (a)–(c)).

**Figure 5:**
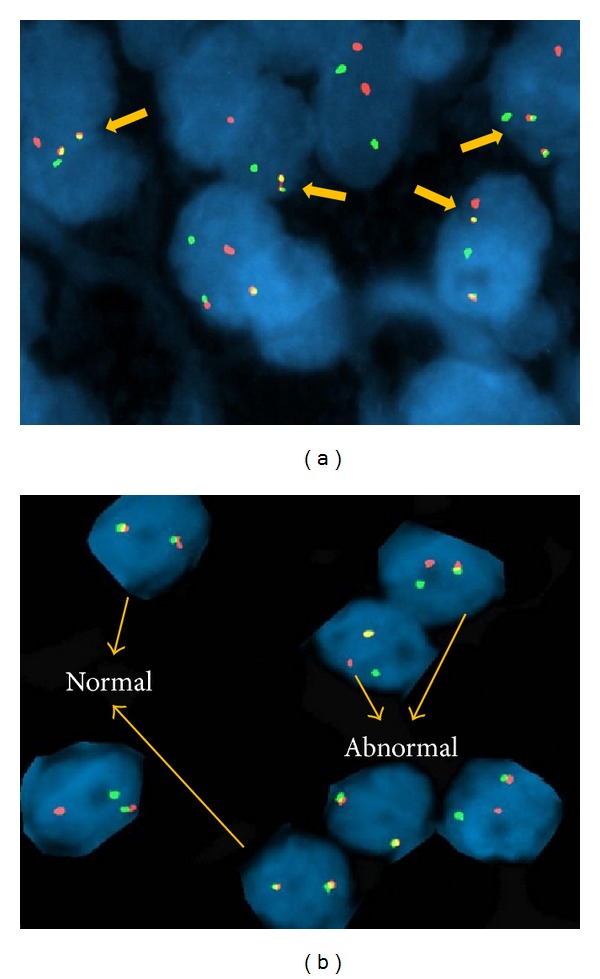
FlSH studies with (a)* API2-MALT1*  fusion signals (yellow) (arrow) with the dual color API2(green)/MALT1(red) dual fusion probe consistent with t(11;18)(q21;q21) and (b) dual color MALT1 “split” probe showing* MALT1* gene rearrangement (arrows-abnormal cells show split signals).

A 44-year-old man with history of colonic diverticulitis presented to the emergency department for evaluation of acute abdominal pain. On physical examination abdominal distention with right lower quadrant abdominal tenderness was recognized. Imaging including abdominal computed tomography (CT) scan with contrast showed small bowel loops distention up to 4.3 cm in diameter and edema with transition point in the mid-lower abdomen concerning moderate enteric obstruction (Figure 1). Furthermore, asymmetric wall thickening involving some of the ileal bowel loops and a few borderline mesenteric lymph nodes was also identified. During laparoscopy, near the transition point, a nonperforated MD was identified at the level of mid-ileum. Macroscopy showed a prominent, inflamed, and indurated MD (Figures 2(a) and 2(b). However, histology showed true diverticulum with intestinal mucosa but failed to show the presence of any ectopic gastric or pancreatic component and microorganism. Furthermore, microscopy revealed extensive infiltration of sheets of small lymphocytes with abundant cytoplasm (monocytoid B-cells) prominently in mucosa and submucosa to form lymphoepithelial lesions (Figures 3(a), 3(b), and 3(c)) and focally transmural involving serosal adipose tissue extending to the surgical resection margins. In some areas, lymphoid proliferation was nodular with occasional reactive germinal centers and expanded marginal zone. The vast majority of the lymphocytes were CD20-positive with weak coexpression of CD43 by immunohistochemical studies (Figure 4(a)). They were also positive for BCL-2 but negative for CD10, Cyclin-D1, and T-cell markers including CD3 and CD5. The germinal centers were reactive, which was confirmed by negative BCL-2 and positive BCL-6 immunohistochemical staining and did not show any evidence of follicular colonization by tumor cells (Figures 4(b) and 4(c)).* In situ* hybridization (ISH)-kappa and lambda showed scattered polyclonal plasma cells and no monoclonal B-cell population was detected. In addition, the proliferation index by ki-67 was less than 5%. The overall morphologic and immunophenotypic features were consistent with ENMZL, low grade. Finally, the fluorescence* in situ* hybridization (FISH) studies revealed* MALT1* gene rearrangement signals pattern in 45.6% of the cells analyzed and was followed by positive API2-MALT1 fusion signals consistent with a reciprocal t(11;18)(q21;q21), in 56.3% of examined cells, confirming the diagnosis (Figure 5). The patient had normal performance status, CBC, and serum basic chemistry panel including LDH and negative HIV serology. Postoperation staging including positron emission tomography (PET) scan and bone marrow biopsy did not show any residual disease or other foci of involvement. However, a few lymph nodes in the lower abdomen demonstrated low level uptake, which may be related to inflammatory changes secondary to the patient's recent surgery or due to involvement by the lymphoma. Therefore, the patient was categorized as low risk International Prognostic Index (IPI), stage I/II An Arbor classification, and stage IIE of Lugano staging system with positive surgical resection margins. Shortly after initial surgery the patient received chemotherapy regimen including Rituximab and Bendamustine. However, because of the proximity of the small bowel, radiation therapy was not being considered as an option. Currently, after 11 months from the initial diagnosis, he has completed 4 cycles of chemotherapy with no complication and his last PET-scan did not show any evidence of recurrence.

## 3. Discussion

Marginal zone lymphoma (MZL) accounts for 5–17% of all non-Hodgkin lymphomas and are classified into three categories: (1) ENMZL of mucosa associated lymphoid tissue (MALT) type which is the most common group, (2) nodal MZL, and (3) splenic MZL [11]. MALT lymphoma is considered as an antigen driven lymphoma associated with prolonged antigen stimulation and is most commonly involved in the stomach followed by salivary glands, orbit, lung, GI tract, breast, thyroid gland, and others [12, 13]. There is often a history of chronic inflammation and antigen stimulation as a result of autoimmune disorder, infection, or other unknown stimuli, which trigger sustained lymphoid proliferation [13, 14]. In our case, the patient had the history of chronic diverticulitis for years. Microscopy usually shows centrocyte-like B-cells (small to medium sized cells with cleaved nucleus and moderately abundant cytoplasm) proliferation with variable number of reactive follicles [15, 16]. Follicular colonization by tumor cells may be seen, which makes it difficult to differentiate it from follicular lymphoma. Transformation to a large cell lymphoma can occur. A central feature of low-grade MALT lymphoma is the presence of lymphoepithelial lesions formed by invasion of individual crypts by aggregates of centrocyte-like cells, which ultimately result in breakdown of the crypt epithelium. Immunophenotypical studies reveal positivity for CD19, CD20, CD22, and CD79a and negativity for CD5, CD23, and BCL-1. The most common cytogenetic abnormality is the reciprocal chromosomal translocation t(11;18), which results in the juxtaposition of the* API2* (apoptosis inhibitor)gene on 11q21, to the* MALT1* gene on 18q21. However, the frequency of API2/MALT1 fusion gene varies among MALT lymphomas originating from different anatomical sites, and little is known about the small bowel MALT lymphoma. The other common chromosomal aberrations include t(1;14)(p22;q32), t(14;18)(q32;q21), and t(3;14)(p14.1;q32) involving* BCL10*,* MALT1*, and* FOXP1*, respectively [17–19]. The molecular genetic testing is helpful to differentiate this type of lymphoma from atypical marginal zone hyperplasia of mucosa-associated lymphoid tissue and immunoproliferative small intestinal disease (IPSID). The former is a benign reactive condition even with lambda or kappa light chain restriction and more common at sites of native MALT such as Peyer patches and tonsils [20]. IPSID also known as alpha chain disease, in the recent World health organization (WHO) classification, is considered as a variant of MALT lymphoma, which is related to* Campylobacter jejuni* as a specific pathogen and most commonly involved small intestine [15, 21]. Another differential diagnosis for GI ENMZL can be any primary GI non-Hodgkin lymphomas such as diffuse large B-cell lymphoma, follicular lymphoma, mantle cell lymphoma (lymphomatous polyposis), Burkitt's lymphoma, and enteropathy associated T-cell lymphoma [22]. However, the histology, immunohistochemical staining, and cytogenetic analyses are usually specific to differentiate these entities from MALT lymphoma. Moreover, another confusing differential diagnosis would be nodal MZL with involvement of GI tract, as patients with a primary GI ENMZL have inferior overall survival in comparison with patients with another ENMZL or nodal MZL [11]. In such a case, Dawson's criteria are useful for labeling primary GI ENMZL lymphoma, which include (1) absence of peripheral lymphadenopathy at the time of presentation; (2) lack of enlarged mediastinal lymph nodes; (3) normal total and differential white blood cell count; (4) predominance of bowel lesion at the time of laparotomy with only lymph nodes obviously affected in the immediate vicinity; and (5) no lymphomatous involvement of liver and spleen [23, 24]. Extensive staging including physical examination, hematological and chemical surveys, CT/PET from cervix to pelvic, and bone marrow aspiration and biopsy is highly recommended for performing appropriate treatment and assessment of prognosis in patients with MALT lymphoma [12]. At presentation, approximately 70–80% of patients with ENMZL present with stage I or II Ann Arbor staging with Musshoff modification [12, 13]. For localized MALT lymphoma, local treatment such as surgical resection or radiation is often curable but the disseminated disease is usually indolent and needs more aggressive treatment. Finally, the 5-year overall survival rate ranged from 76% to 98% in different studies and depends on age, primary site of involvement, stage, and international prognostic index [11, 12, 25, 26]. We report this case to emphasize that neoplastic process like extranodal marginal zone lymphoma can occur in MD, and this is the first such reported case. In such cases, the patient should undergo more evaluation, aggressive therapy, and closer follow-up.
